# Temporal and geographic patterns of kinship structure in common dolphins (*Delphinus delphis*) suggest site fidelity and female-biased long-distance dispersal

**DOI:** 10.1007/s00265-017-2351-z

**Published:** 2017-07-21

**Authors:** Laura Ball, Kypher Shreves, Małgorzata Pilot, André E. Moura

**Affiliations:** School of Life Sciences, University of Lincoln, Lincoln, UK

**Keywords:** Kinship, Social structure, Common dolphin, Cetacea

## Abstract

**Abstract:**

Social structure plays a crucial role in determining a species’ dispersal patterns and genetic structure. Cetaceans show a diversity of social and mating systems, but their effects on dispersal and genetic structure are not well known, in part because of technical difficulties in obtaining robust observational data. Here, we combine genetic profiling and GIS analysis to identify patterns of kin distribution over time and space, to infer mating structure and dispersal patterns in short-beaked common dolphins (*Delphinus delphis*). This species is highly social, and exhibits weak spatial genetic structure in the Northeast Atlantic and Mediterranean Sea, thought to result from fluid social structure and low levels of site fidelity. We found that although sampled groups were not composed of closely related individuals, close kin were frequently found in the same geographic location over several years. Our results suggest that common dolphin exhibits some level of site fidelity, which could be explained by foraging for temporally varying prey resource in areas familiar to individuals. Dispersal from natal area likely involves long-distance movements of females, as males are found more frequently than females in the same locations as their close kin. Long-distance dispersal may explain the near panmixia observed in this species. By analysing individuals sampled in the same geographic location over multiple years, we avoid caveats associated with divergence-based methods of inferring sex-biased dispersal. We thus provide a unique perspective on this species’ social structure and dispersal behaviour, and how it relates to the observed low levels of population genetic structure in European waters.

**Significance statement:**

Movement patterns and social interactions are aspects of wild animal’s behaviour important for understanding their ecology. However, tracking these behaviours directly can be very challenging in wide-ranging species such as whales and dolphins. In this study, we used genetic information to detect how patterns of kin associations change in space and time, to infer aspects of movement and social structure. We identified previously unknown site fidelity, and suggested that dispersal usually involves females, travelling long distances from the natal area. Our data analysis strategy overcomes known limitations of previously used genetic inference methods, and provides a new approach to identify differences in dispersal between the sexes, which contribute to better understanding of the species’ behaviour and ecology. In this case, we suggest that females are more likely to disperse than males, a pattern unusual amongst mammals.

**Electronic supplementary material:**

The online version of this article (doi:10.1007/s00265-017-2351-z) contains supplementary material, which is available to authorized users.

## Introduction

In social animals, patterns of social interactions and kin association are determined by a combination of intrinsic and extrinsic factors, such as intra-specific competition, dispersal behaviour, predator avoidance and food availability. Social structure and dispersal patterns can also be linked with specific mating strategies, often resulting in sex-biased dispersal (Clutton-Brock and Lukas 2012). Therefore, insight into the interactions between kin association patterns, social structure and mating systems of wild animals can provide important information on their ecology, demographic structure and dispersal behaviour.

In highly mobile species that can travel over large distances, dispersal patterns are difficult to assess directly. Although individual movements can be tracked through tagging, radiotelemetry or satellite telemetry, they do not provide information on population-level dispersal patterns. Alternatively, this information can be inferred from patterns of genetic variability, assessed at the level of social groups and entire populations. For example, genetic studies of grey wolf (*Canis lupus*) populations revealed that dispersal of individuals is biased towards habitats similar to their natal habitats (Pilot et al. 2006; Musiani et al. 2007), which was previously unknown despite extensive ecological research on this species. Further, genetic studies on primates revealed that dispersal is mostly male-biased, with some exceptions such as chimpanzees (*Pan troglodytes*), bonobos (*Pan paniscus*) and hamadryas baboons (*Papio hamadryas*), where dispersal is female-biased (reviewed in Vigilant and Guschanski 2009).

Knowledge on dispersal patterns, mating systems and composition of social groups in cetaceans is limited due to difficulties with obtaining robust behavioural data in the marine environment. In several well studied species, such as bottlenose dolphins (*Tursiops* spp.) and killer whales (*Orcinus orca*), this knowledge has been obtained mostly from population genetic studies (reviewed in Möller 2012). All species of toothed cetaceans are known to form social groups, often grouping for the purposes of migration, protection, feeding and reproduction (Connor 2000). The size and social structure of these groupings can vary greatly between species (reviewed in Möller 2012) and in some cases even within species, as seen in killer whales and bottlenose dolphins (Ford et al. 2000; Connor and Krützen 2015). Such differences are thought to be determined by a combination of factors, including the availability of predictable prey resources (Möller 2012) and the risk of predation in open-water environments (Gowans et al. 2007). These are thought to affect patterns of kin association, but information is still lacking for many cetacean species (Möller 2012).

In some odontocetes, social groups tend to consist of individuals sharing close kinship relationships. Matrilineal pods, where a breeding female retains close association with its offspring, are seen in resident killer whales (*O. orca*; Ford et al. 2000), short-finned pilot whales (*Globicephala macrorhynchus*; Heimlich-Boran 1993) and female sperm whales (*Physeter macrocephalus*; Lyrholm and Gyllensten 1998). However, in other cetacean species such as Commerson’s dolphins (*Cephalorhynchus commersonii*; Coscarella et al. 2011) or Tucuxi (*Sotalia guianensis*; Santos and Rosso 2008), such close kin associations are not always present or occur only transiently (Connor 2000).

The social behaviour of bottlenose dolphins (*Tursiops* spp*.*) is a well-documented example of this type of social structure. This genus is characterised by a fission-fusion structure, where factors such as age, sex and reproductive status likely influence the social strategies of each individual dolphin (Connor 2000). Relatedness has also been identified as a factor, with female *Tursiops aduncus* in Eastern Australia showing preferred association with related females (Möller et al. 2006; Wiszniewski et al. 2010), whilst *Tursiops truncatus* in the Bahamas display greater frequency of male alliances between related individuals than would be expected by chance (Parsons et al. 2003). In spite of these well described cases, such effects of kinship on social association patterns have not been universally observed across populations of *Tursiops* spp*.* (Connor 2000; Möller 2012).

Common dolphins (*Delphinus* spp.) are highly social and are often seen in groups ranging from less than 30 to thousands of individuals (Perrin 2009). Information on dispersal patterns and site fidelity is extremely scarce, although genetic structure suggests potential long-range movements (Moura et al. 2013), supported by a single photo-ID observation (Genov et al. 2012). Scientific literature regarding its social structure and mating system is also scarce, with an earlier study suggesting it to be matrilineal (Amos 1999). More recently, a fission-fusion structure similar to bottlenose dolphins has been described (Bruno et al. 2004; Zanardo et al. 2016), which is consistent with the expectations for small pelagic delphinids (Möller 2012). Relative testis size (Murphy et al. 2005) indicates strong sperm competition, which is consistent with a more fluid social structure and low kinship association.

Short-beaked common dolphins in the Northeast Atlantic show low levels of genetic structure (Natoli et al. 2006; Mirimin et al. 2009; Moura et al. 2013), which also suggests limited kin clustering and high levels of gene flow. On a global scale, significant differentiation was found between populations inhabiting different ocean basins (Natoli et al. 2006), which was attributed to isolation by distance (Amaral et al. 2012). In certain regions of the eastern and southern Australian coasts, short-beaked common dolphins display fine-scale genetic structure (Bilgmann et al. 2008, 2014; Möller et al. 2011), which may imply differences in kin association patterns between regional populations.

A genetic study on kinship levels between individuals from a single mass stranding of common dolphin (*Delphinus delphis*) showed no evidence for kinship structure within a group (Viricel et al. 2008). However, mass-stranding events may not be representative of the natural social grouping of the species. In fact, a study on pilot whales (a species for which mass strandings are more frequent than for common dolphin) has revealed that the social structure inferred from such events differs strongly from that inferred from free-ranging groups in this species (Oremus et al. 2013).

Assessing kinship relations of common dolphins in their natural environment is thus required to gain new insight into the social organisation of the species. The frequency of first-, second- and third-degree relatives within social groups found in the wild may be used to further clarify common dolphin social structure and dispersal patterns. A recent study found that kinship could be an important determinant in group composition in Australian short-beaked common dolphin, particularly between males (Zanardo et al. 2016). Because kin association in dolphins can be transient, data from different time periods and locations are required to avoid context-dependent bias.

In this study, we provide an assessment of social structure in short-beaked common dolphin (*D. delphis*) based on free-ranging animals along the Portuguese coast, which are part of the larger Atlantic population where no studies on social structure have been carried out. Biopsy samples were obtained from multiple groups found in the same locations during multiple years, and kinship patterns were identified using individual multilocus genotypes. Kinship patterns were then analysed in the context of group composition, geographic location and year of sampling. We specifically assessed whether (1) groups of interacting common dolphin individuals are composed of related individuals, (2) individuals are found in the same locations as their close kin over multiple years and (3) kinship level between individuals increases with geographic proximity of locations where they are observed.

If the lack of genetic differentiation in European common dolphins is due to high promiscuity, with an associated fluid social structure and low philopatry, then no geographical patterns of kinship should be detected. Therefore, analysing the spatial and temporal distribution of kinship associations will allow us to not only improve our understanding of common dolphin social structure and dispersal patterns, but also shed light on how social structure shapes genetic structure in wild mammals. It will also evaluate the potential of genetic-based kinship analyses in inferring demographic and social patterns from elusive wild animals, for which field data might be difficult to obtain.

## Methods

### Sampling of wild dolphins

A total of 204 biopsy samples were collected from free-ranging, short-beaked common dolphins (*D. delphis*) in five separate locations along the Portuguese coast, separated by approximately 20 to 100 nautical miles. Samples were collected throughout three separate field seasons (2007–2009), although not all locations were sampled in all of the seasons (Table 1). Sampling was carried out with permits from the Portuguese Institute for the Conservation of Nature (ICNB) as described in (Moura et al. 2013). Importantly for this study, only animals that were clearly of adult size were sampled, and no animals accompanied by calves were targeted, so no bias in relatedness estimates is introduced from sampling mother-calf pairs. We cannot exclude that sexually immature individuals of adult size could have been collected alongside sexually mature adults. Sub-adults may display different kin association patterns as compared to adults (Mason et al. 2016), but in this study, it was not possible to assess age-specific kin structure.

**Table 1:** Number of biopsy samples of short-beaked common dolphins (*Delphinus delphis*) genotyped for 15 microsatellites in this study, for each location and year of sampling

	2007	2008	2009	Total
Porto	–	12	14	26
Figueira	–	16	10	26
Peniche	–	30	–	30
Sines	2	19	10	31
Sagres	19	0	21	40
Portimão	13	10	28	51
Total	34	87	83	204

We considered all individuals sampled in the same sampling event as belonging to the same group, irrespectively of the behaviour exhibited. A new sampling event was considered whenever more than 1 h had elapsed from the last biopsy sample taken from the previous group. This was the only criterion used to define groups, given that lack of knowledge of the social behaviour typical of this species locally, precluded accurate identification of social groups if sampling was interrupted for longer. Sampled groups ranged from approximately 20 to several hundred individuals, but exact numbers for each sampling event were not recorded. The sampling permit introduced a limit of ten samples per group to minimise animal welfare impact from the biopsy sampling effort. Within this limitation, each group was sampled as inclusively as possible to allow assessment of kinship within groups and to avoid bias from sampling potential nursing groups. It was not possible to record data blindly because our study involved focal animals in the field.

### Kinship analyses

All individuals were genotyped for 15 microsatellite loci as described previously (Moura et al. 2013), and sex was determined using the ZFX/ZFY protocol (Bérubé and Palsbøll 1996). Estimating kinship from microsatellite genotypes alone may lead to misclassification of kinship relationships, if based on a small number of loci or if the loci analysed have low genetic variability (Blouin et al. 1996). However, moderate numbers (12–20) of highly variable loci provide sufficient resolution to be used as legal evidence (e.g. in assignment of human paternity or in individual identification) and were shown to accurately identify known kinship relationships in wild animals (e.g. Pilot et al. 2010b). To assess whether levels of genetic variation in our study population were high enough to achieve sufficient accuracy of kinship estimates, observed and expected heterozygosity were estimated for each locus individually and for overall loci, as well as for average individual heterozygosity, all calculated using GENALEX (Peakall and Smouse 2006). Furthermore, probability of identity as well as probability of no exclusion of parents and siblings were calculated in CERVUS (Kalinowski et al. 2007).

We identified two samples, collected in the same location but in different years, with an identical genotype, implying that the same individual was sampled twice. We kept the genotype from both samples when we assessed kinship relationships within groups, but removed the genotype of one sample for the comparisons between groups.

Individual’s parentage and kinship was estimated using the maximum-likelihood approach, which accounts for genotyping errors, implemented in the software packages CERVUS and KINGROUP (Konovalov et al. 2004). Because no prior information on social structure is available for the study population, only adults were sampled and their age was unknown; all individuals were considered as both candidate parents and offspring. Therefore, we applied a rigorous data analysis strategy to minimise the chances of obtaining false positives, as described in earlier studies (Pilot et al. 2010a; Moura et al. 2014).

Three parentage tests were performed in CERVUS: one to identify potential mother-offspring pairs, considering only the females as potential parents; another to identify potential father-offspring pairs, considering only the males as potential parents; and a last one to identify parent pairs, where males and females were considered as potential parents simultaneously. Confidence levels were estimated by simulating populations with known parent-offspring pairs, based on allele frequencies of the real population genotypes. Simulations assumed 10,000 offspring, 83 mothers and 122 fathers, corresponding to the number of males and females in the dataset (they were all adults and therefore potential parents). Simulations also assumed that 80% of parents of both sexes remain unsampled. We considered two confidence levels—strict (95%) and relaxed (80%).

In some cases, the parent-offspring pair was identified twice with individual parent-offspring assignment reversed. Because we did not know the age of individuals, it was impossible to know which of these assignments was correct, but for our purposes, it was not necessary to know which individual was a parent and which was an offspring. When such reverse parent-offspring assignments occurred, only one of the results was kept and the match noted. If no parentage was assigned to an individual at either strict or relaxed confidence level, then it was concluded that no parents or offspring of a given individual were sampled.

KINGROUP was used as a complementary method of identifying groups of close kin. We used this program to identify the most likely partition of all individuals into groups of kin related at a given level (e.g. full-siblings, half-siblings). The full sibship reconstruction (FSR) method was implemented, which partitions the analysed sample set into groups of kin sharing a common relationship. This allows for single-individual groups, which represent those individuals that do not have any relatives at the particular level (e.g. full-siblings), within the dataset. The most likely partition into kin groups is determined by calculating the likelihood that individuals in a group share a hypothesised kinship relationship (the primary hypothesis). Simulations are then used to test if that relationship level is significantly more likely than an alternative relationship (the null hypothesis). Because multiple alternative relationships are often possible, complex null hypotheses were tested. The primary hypothesis that the group consists of full-siblings was tested against the null hypothesis of the relationship at the level of half-siblings to unrelated. The primary hypothesis that the group consists of half-siblings was tested against the null hypothesis of the relationship at the level of cousins to unrelated.

Using this method, we reconstructed the partition of the analysed dataset into three kinship classes—full-siblings, half-siblings and cousins. KINGROUP has a limited ability of distinguishing between parent-offspring and full-siblings, so CERVUS parent-offspring pairs were accepted if KINGROUP found them to be related at either a parent-offspring or a full-sibling level.

Because KINGROUP identifies groups of individuals at a particular level of relatedness, full-sibling groups may also include parent-offspring pairs (with the same expected proportion of 50% shared alleles), and half-sibling groups may include, e.g. grandparent-offspring pairs (with the expected proportion of 25% shared alleles). Parent-offspring pairs were distinguished from full-sibling pairs by including an additional condition that they have to share at least one allele at each locus (full-siblings share 50% alleles at average, but may not share any alleles at some individual loci).

When comparing the two parents-offspring trio matches to establish the likelihood of an assigned parent being correct, the number of mismatching loci and pair confidence were considered. The putative parent with the least mismatching loci and an assigned confidence level was predicted to be the most likely parent.

### Patterns of kin group occurrence and distribution

Kin relationships were then integrated with information regarding sampling group, time of sampling and location of sampling. Specifically, we were interested in determining which of those factors maximised the grouping of related individuals. It should be noted that because of how groups were defined (see earlier), individual groups might have been sampled multiple times in the same day, if found at discrete times of the day.

We determined the number of kin groups including individuals sampled in the same day, month and year for each of the three different kinship relationships independently, and compared them to groups containing individuals sampled across different years. The number of kin groups containing individuals sampled exclusively in the same location, was also compared to the number of kin groups containing individuals sampled in different locations. When a group was found to be sampled only in two different locations, we discriminated whether these locations were geographically next to each other or not. Correlation between the number of kin groups and the location was assessed through contingency table analyses, using a Pearson chi-squared test and a Fisher exact test (in tables small enough for computations) using the software PAST (Hammer et al. 2001). These analyses were repeated separately for males and females in the parent-offspring category to test for potential sex-biased dispersal from natal group. Further tests for sex-biased dispersal were performed in the FSTAT package (Goudet 2001; Goudet et al. 2002) by comparing the sex-specific mean Assignment Index (mAIc), variance of Assignment Index (vAIc), pairwise *F*_ST_ between locations, mean *F*_IS_ and mean pairwise relatedness. If sex-biased dispersal is occurring, then the dispersing sex is expected to have lower mAIc, *F*_ST_, *F*_IS_ and mean pairwise relatedness, but higher vAIc.

For cousin groups inferred in KINGROUP, we calculated the proportion of each group that was found in a given location, and represented the proportion geographically. This was done by defining the four points representing the maximum extent of sampling at each location, and performing ordinary krigging interpolation for each cousin group proportion at a given location, as implemented in the ArcMap (ESRI) geographic information system. This was done only for cousin groups, which reflect wider family relationships and therefore are more appropriate to assess the population level extent of natal dispersal. Furthermore, kin groups of closer relationships were usually composed of too few individuals to allow robust interpolation.

## Results

### Parentage and kinship analysis

In this study, parentage and kinship in the sampled population were inferred solely based on individual microsatellite genotypes, which require high levels of genetic variation (see “Methods” section for more details). In the population studied here, the probability of identity based on the 15 microsatellites was very low (PI = 7.23 × 10^−18^), as were all probabilities of non-exclusion of parents/siblings (all below 1.3 × 10^−4^). The heterozygosity levels across all loci were high (*H*_E_ = 0.72, *H*_O_ = 0.72, *H*_Ind_ = 0.72), consistent with the heterozygosity levels described in other studies for common dolphin in the eastern North Atlantic (*H*_E_ = 0.76–0.82, *H*_O_ = 0.69–0.74, Natoli et al. 2006; *H*_E_ = 0.705, *H*_O_ = 0.695, Mirimin et al. 2009), though note that not all the same loci were used across studies. Two of the 15 microsatellite loci scored had *H*_E_ lower than 0.2 (KWM1b and TexVet9). After excluding those two loci, mean *H*_E_ and *H*_O_ were both 0.81. Both values (0.72 and 0.81) are within the ranges identified as giving low rates of kinship misclassification (~5%) in earlier simulation studies (Blouin et al. 1996). Furthermore, although early methods of kin identification were based on estimating pairwise relatedness without assessing confidence levels of inferred relationships (Blouin et al. 1996; Van Horn et al. 2008), here we used methods of parentage and kinship analysis that estimate the likelihood of each inferred relationship. This provides an efficient way to minimise the occurrence of misclassified relationships.

The maximum-likelihood approach implemented in CERVUS 3.0 (Kalinowski et al. 2007) assigned a single parent to 68 individuals overall, with 39 being fathers and 29 being mothers. For 12 individuals, CERVUS assigned both parents. In addition, 30 other parent-offspring matches consisted of 15 pairs of matches where it was impossible to determine which individual was the parent and which was the offspring (as both relationships were assigned high confidence). Of all the assigned parents, three females were assigned as mothers to more than one offspring, and four males were assigned as fathers of two or more offspring.

Of all single parent assignments, seven fathers and six mothers were assigned with strict confidence (95%), whilst 25 fathers and 21 mothers were assigned with relaxed confidence level (80%). Of all parent-pair assignments, two pairs were assigned with strict confidence, and eight pairs were assigned with relaxed confidence. Although pairs assigned with relaxed confidence are more likely to be false positives, they are likely to represent close kin relationship other than parent-offspring pairs, such as full-siblings.

**Fig. 1:**
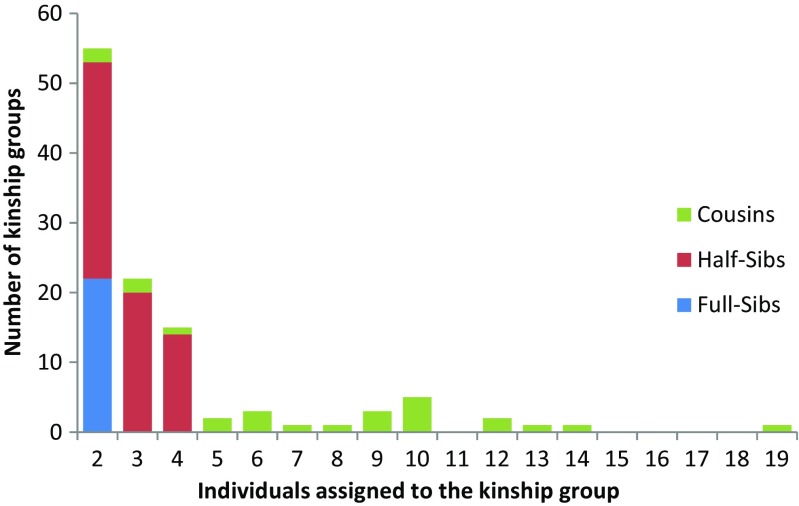
Frequency distribution of the number of individuals of short-beaked common dolphin (*Delphinus delphis*) belonging to different kinship classes, as inferred with KINGROUP

KINGROUP inferred a total of 22 full-sibling groups, 65 half-sibling groups and 25 cousin groups. Comparison with CERVUS results allowed six out of the 22 full-sibling pairs to be identified as parent-offspring matches (Table S1). Full-sibling groups consisted of two individuals, with half-sibling and cousin groups ranging from 2 to 4 and 2 to 19 individuals respectively, with 47% of half-sibling groups consisting of only two individuals (Fig. 1).

### Temporal distribution of kin groups

Overall, our study sampled 46 different groups of interacting individuals, with an average of 3.9 sampled individuals per group (see Fig. S1 for the frequency distribution of group sample number). Individuals with close kinship relationships (parent-offspring, full-siblings and half-siblings) were never sampled in the same group, and 14 pairs of individuals related at the cousin level were sampled in the same group. No offspring were sampled on the same day or month as either of their parents, but they were frequently sampled in the same year (Table 2). On the other hand, one pair of full-siblings and several groups of half-siblings and cousins, included individuals that were sampled on the same day (Table S2). Regardless, most relatives were sampled in different years. For example, only 40% of the full-sibling groups were sampled in the same year (Table 2). Although the majority of half-sibling and cousin groups were composed of individuals sampled across different days/months, they often included individuals that were sampled in the same day/month. For example, cousin group 13 contained two individuals sampled on 28 August 2008, two sampled on 06 August 2009 and another two on 16 August 2009 (Table S2). The half-siblings and cousins sampled together at the same time, were all from kin groups larger than two individuals. This means that other members of the kin group were sampled in different social groups, and in many cases, they were sampled in different months or years.

**Table 2:** Temporal pattern of sampling of kin from the groups of short-beaked common dolphins (*Delphinus delphis*) along the Portuguese coast

Sampled on the same	Number of kin groups/pairs						
	Mother-offspring	Father- offspring	Full-siblings	Half-siblings		Cousins	
				Whole group	Containing individuals	Whole group	Containing individuals
Day	0	0	1	1	6	0	13
Month	0	0	2	3	14	0	22
Year	17	21	7	13	26	0	22
None	12	18	15	23	29	3	22

The table shows the number of different kin groups that included individuals sampled within the same time classes (the same day, month or year). ‘Whole group’ refers to groups where all the individuals were sampled in the specific time class, whilst ‘containing individuals’ refers to groups where only some individuals were found in the specific time class. Note that grouping in time classes is hierarchical; e.g. the number of individuals sampled in the same month also includes individuals sampled in the same day

### Geographic distribution of kin groups

Overall, 16 parent-offspring pairs (as identified in CERVUS) were sampled in the same location (Table 3). The remaining 52 parent-offspring pairs were found in multiple locations, in different combinations between the six locations. For parent-offspring pairs, no mothers and only four fathers were sampled in the same location as the offspring, whilst none of the mother-father pairs were sampled in the same location (Table 3).

**Table 3:** Numbers of parent-offspring pairs and sex ratio in the short-beaked common dolphin (*Delphinus delphis*) sampled in different locations along the Portuguese coast across all years of the study

Location	Total males sampled	Total females sampled	Ratio (M/F)	M-O pairs	F-O pairs	Trio matches			Overlapping pairs	
						M-O	F-O	M-F	M-O	F-O
Porto	14	12	1.16:1	0	0	0	0	0	0	0
Figueira	15	11	1.36:1	0	1	0	0	0	0	1
Peniche	20	10	2:1	1	2	0	0	0	0	1
Sines	25	6	4.17:1	0	2	0	0	0	0	2
Sagres	18	22	1:1.22	0	0	0	0	0	0	0
Portimão	29	22	1.32:1	5	5	0	4	0	2	2
Multiple	–	–	–	23	29	12	8	12	9	13

Parent-offspring pairs were identified using CERVUS software. ‘Overlapping pairs’ are those for which CERVUS could not determine which individual was the parent or the offspring

*F* female, *M* male, *O* offspring

Generally, kin groups that were composed of more individuals tended to have a larger geographical area where members of the kin group were sampled (Table S2). Only three of the 31 half-sibling groups consisting of two individuals were sampled in the same area. Half-sibling and cousin groups often consisted of more than two individuals, and therefore, their distribution could be spread out over multiple locations.

Most kin groups identified in this study (independent of their size) were found in two or less locations (across all years); these could be either two neighbouring locations or two separate locations (i.e. locations that were not situated next to each other). Correlation between the number of kin groups and sampling location was significant (*χ* ^2^ = 18.233, *p* = 0.019), with most kin groups at a level higher than half-siblings (53%) found in the same or neighbouring locations relative to separate locations (although the difference was higher for father-offspring groups; Table 4).

**Table 4:** Frequency of different kinship classes found in groups of short-beaked common dolphins (*Delphinus delphis*) across sampling locations along the Portuguese coast

Sampling locations	Number of kin groups								
	Mother-offspring		Father-offspring		Full-siblings			Half-siblings	Cousins
	F	M	F	M	F	M	B		
Same	2	4	4	6	1	3	2	4	0
Neighbouring	4	3	3	10	3	1	2	17^a^	8^a^
Separated	5	11	13	3	1	4	5	44^a,b^	17^a,b^
Total	11	18	20	19	5	8	9	65	25

Kinship classes were identified using CERVUS and KINGROUP

*F* female, *M* male, *B* male-female pairs

^a^Some individuals sampled in the same location

^b^Some individuals sampled in neighbouring locations

When these analyses were repeated for parent-offspring pairs considering male and female offspring separately, a significant correlation was found between the number of kin groups and the sampling location for male offspring (*χ* ^2^ = 10.446, *p* = 0.033; Fisher’s *p* = 0.0297) but not for female offspring (*χ* ^2^ = 5.045, *p* = 0.283; Fisher’s *p* = 0.24). Fewer mothers were found in the same region as their offspring compared to fathers and their offspring (six vs ten pairs, respectively). Consistently, less female offspring were found in the same location as one of their parents than male offspring (six vs ten, respectively). For male offspring, most individuals were found in the same or neighbouring locations as their fathers, but in separate locations to their mothers (Table 4).

More full-sibling groups were found in separate locations than in the same or neighbouring locations (Table 4). Of those found in the same locations, more brothers and brother-sister pairs were sampled in the same location, compared with sister pairs. More brothers were sampled in the same location, whilst more sisters were sampled in neighbouring locations. However, these differences are marginal, as very few full-sibling groups were inferred. Amongst half-siblings and cousins, there were groups that included both individuals sampled in the same locations and individuals sampled in separate locations. In the case of half-siblings, four groups contained individuals that were all sampled in the same area (Table 4).

**Fig. 2:**
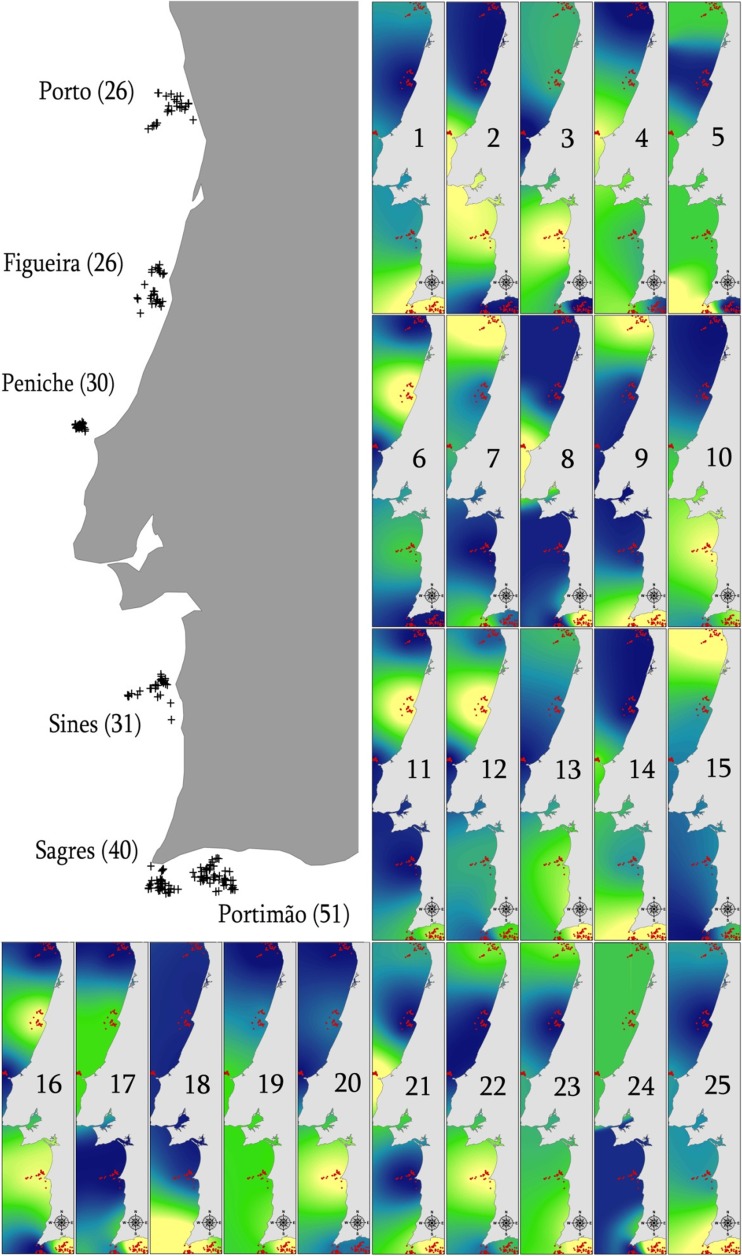
Spatial distribution of biopsy samples and cousin groups of short-beaked common dolphins (*Delphinus delphis*) along the Portuguese coast. *Numbers in brackets* represent the sample size for each location. *Yellow colour* represents a high proportion of all individuals from that particular group, whilst *blue colour* represents no individuals from the group. *Numbers 1–25* refer to different inferred cousin groups. Maps are not to scale; however, distance between the centres of Sagres and Portimão sample points is approximately 35 km

Only one group of cousins had members which were found in all sampled areas. Cousin groups were more commonly split between four areas (40% of all cousin groups); however, the geographical distribution of these groups shows that even in such cases, most individuals will be concentrated in only one or two areas (Fig. 2; e.g. groups 1, 2 and 10). These areas were often contiguous, but some cousin groups were separated by large geographic distances, which might include one or more locations where no members of that group were found (Fig. 2; e.g. groups 7, 8, 15 and 17). All classic sex-biased dispersal tests carried out were non-significant (Table S3), which would suggest no sex-biased dispersal, though note the discussion below regarding the applicability of such tests to this dataset.

## Discussion

### Common dolphin social structure

The results of this study show that common dolphin social groups do not consist of closely related individuals, which is consistent with a previous study on mass strandings (Viricel et al. 2008). This contrasts with the kin-based social structure seen in other delphinids such as killer whales (Ford et al. 2000; Pilot et al. 2010b) or pilot whales (Heimlich-Boran 1993). The pattern observed in the common dolphin fits the expected pattern for small delphinids, where association of unrelated individuals is expected to occur due to reliance on unpredictable prey resources, as in such conditions there would be no benefit for either sex to exhibit any degree of philopatry (Möller 2012).

However, social groups that do not consist of close kin are relatively rare in mammals, and even in cases where social grouping is not based on kin association, such as gorillas, sub-groups of close kin within a group can be detected (Bradley et al. 2007). It cannot be excluded that the common dolphin groups also contain sub-groups of close kin, which remain undetected because our sample size was small relative to the size of the sampled groups. The geographic distribution of kin groups observed in our study suggests a certain degree of natal philopatry in common dolphins, with most groups containing kin found in the same or neighbouring location. This is consistent with a fission-fusion structure (Bruno et al. 2004), particularly such as reported for bottlenose dolphin (Connor and Krützen 2015). It should be noted that, given the short distance between sampling locations relative to the species’ dispersal abilities and known genetic panmixia across Europe for this species (Moura et al. 2013), groups found in neighbouring locations still likely reflect fidelity to a large home range, rather than dispersal from natal area.

The spatial distribution of kin inferred in our study differed between the sexes. Male kin were mostly found in the same or neighbouring locations (implying limited male dispersal), but female kin were mostly found in non-neighbouring, geographically distant locations, indicating that females disperse over longer distances than males on average. This result is consistent with those found in Zanardo et al. (2016), where kinship between individuals within a pod in common dolphins was higher for males than females.

Although our kinship analysis suggests a degree of female-biased dispersal in Northeast Atlantic common dolphins, other methods used gave non-significant results, which is consistent with previous results for this species on a larger scale (Natoli et al. 2008). However, methods based on sex-specific *F* statistics and assignment values, have low power in systems where the sex dispersal asymmetry is low and overall dispersal rates are high (Goudet et al. 2002). Common dolphins in Europe are panmictic (Moura et al. 2013), which results in very low pairwise *F*_ST_ values between locations. Therefore, genetic differences between immigrants and phylopatric individuals will be low, which greatly reduces the power of classical sex-biased dispersal tests (Goudet et al. 2002). Therefore, we suggest that comparison of kin groups over space and time remains, for the moment, the most accurate method to assess sex-biased dispersal in European common dolphins, where sex-specific asymmetries in dispersal are likely to be subtle.

### Potential drivers of common dolphin kinship and dispersal patterns

Inbreeding avoidance has been proposed as a potential mechanism promoting sex-biased dispersal (Perrin and Mazalov 2000; Lawson Handley and Perrin 2007; Clutton-Brock and Lukas 2012). The lack of association between parents and offspring found in this study may result from inbreeding avoidance in groups with a fluid social organisation. Female-biased dispersal as an inbreeding avoidance mechanism is more likely to develop in polygynous mating systems (Clutton-Brock 1989; Perrin and Mazalov 1999), but there is no evidence for common dolphins having such a mating system, and this is not supported by our results either.

Although fission-fusion social structures involve the creation of male alliances to maintain access to females in some bottlenose dolphin populations (Connor et al. 1992; Möller et al. 2001; Parsons et al. 2003), this has not been reported for common dolphins. Furthermore, relatively large testis size (Murphy et al. 2005; MacLeod 2010) suggests that females copulate with several males. Field observations are consistent with this, as during sampling efforts, we commonly observed a mating behaviour where a female allows several competing males to follow her until one male is chosen for copulation, although such behaviours have not been described in other small delphinid species.

It is thus likely that females mate with multiple males, in which case the mating system in common dolphins might be better described as polygynandrous (Watts 1998; Díaz-Muñoz et al. 2014). In such mating systems, sex-biased inbreeding avoidance strategies are more likely to result in male-biased dispersal (Dobson 1982; Lawson Handley and Perrin 2007). Given that the evidence presented here is more consistent with a certain degree of female-biased dispersal, we instead suggest that sex-biased dispersal observed in this study may reflect local ecological factors specific to this population.

Male-biased dispersal appears to be the norm in mammals (Greenwood 1980; Lawson Handley and Perrin 2007) and is largely thought to represent their ancestral state (Clutton-Brock and Lukas 2012). Nevertheless, cases of female-biased dispersal have also been described in mammalian species, such as the greater sac-winged bat (Nagy et al. 2007) and porcupines (Sweitzer and Berger 1998). For some species, such as the red deer, both male- (Catchpole et al. 2004) and female-biased (Pérez-González and Carranza 2009) dispersal have been suggested. In cetaceans, male-biased dispersal has been inferred only in species that inhabit environments where prey resources are predictable (Möller 2012), whilst species living in unpredictable environments have symmetrical sex dispersal. This includes the common dolphin, although the above discussion of the low power of sex-specific *F*_ST_ and assignment methods should be noted.

Theoretical models suggest that, in promiscuous mating systems, female-biased dispersal is likely to develop if local resource competition affects female reproductive success (Perrin and Mazalov 2000). When common dolphins hunt, the process of aggregation of fish schools requires cooperation between individuals. Once the fish is trapped against the surface, individual dolphins might then compete for access to the disoriented fish. Studies on common dolphin feeding ecology show that females and juveniles often exhibit different feeding strategies to adult males (Silva 1999; Nino-Torres et al. 2006). In a scenario of competitive access to food, juveniles and females can be at a disadvantage, as their smaller size makes them less able to compete (Ruckstuhl and Neuhaus 2000; Meynier et al. 2008).

In these circumstances, during periods of reduced prey abundance, females could have reduced access to prey due to increased competition with larger males, particularly if the female is pregnant or accompanied by a calf. Therefore, females benefit the most from dispersing elsewhere, where prey may be more abundant and local resource competition is less intense. The only study so far available on common dolphin dispersing behaviour reports the long-distance dispersal of a female dolphin accompanied by a calf, which was followed by site fidelity to new area for at least 1 year (Genov et al. 2012). Long-distance dispersal means here that the animals do not expand their natural range continuously, but ignore some close-by locations with suitable habitats and settle farther apart.

A calf accompanying a dispersing female could be a male, in which case male dispersal would also occur. Given a sex ratio of 1:1, this would effectively skew dispersal towards females, as only half of the dispersal events would statistically be expected to involve a male. This would be consistent with the idea discussed above that sex dispersal asymmetry, if present, is likely to be subtle in this system.

### Kinship patterns and large-scale panmixia

It thus appears that the fluid social structure in short-beaked common dolphins and the genetic panmixia over large spatial scales in the Northeast Atlantic and the Mediterranean Sea may result from their specific feeding ecology. Common dolphins feed mainly on high-energy epipelagic schooling fish (Spitz et al. 2010), through a technique that requires cooperation between large numbers of individuals. This technique thus promotes social interactions between individuals irrespective of their level of relatedness. Distribution of such prey items tends to be seasonal and is dependent on specific environmental characteristics, making its exact distribution variable in time. In the Portuguese coast, common dolphin’s preferred prey is the sardine (*Sardina pilchardus*; Silva 1999), whose patterns of abundance and distribution are known to fluctuate between years (Santos 2001; Borges et al. 2003). Some regions might have more favourable conditions, and therefore, the appearance of suitable prey can be predictable to some extent. This increases the costs of exploring larger unfamiliar areas where prey abundance patterns are uncertain. This can lead to habitat dependence and natal philopatry, but also promote the long-distance dispersal behaviour when local prey resources become depleted.

## Conclusions

The results of this study show that common dolphin (*D. delphis*) large-scale panmixia in Europe (Moura et al. 2013) likely results from long-distance dispersal of individuals questing for seasonal prey, rather than from extreme promiscuity and fluid social structure. Our results provide new insight into the social and mating systems of this poorly studied species, for which field observation data on mating behaviour are scarce. Our analysis of the temporal and geographic distribution of kin groups provided support for a fission-fusion social structure in common dolphins. Furthermore, this analysis indicates female-biased dispersal, which had so far remained undetected. This dispersal pattern is consistent with the occurrence of competition for an unpredictable prey resource and the lack of paternal care in this species. Female-biased dispersal is uncommon in mammals, and therefore, more research based on a larger sample size is needed to confirm this pattern. Despite the limitations resulting from relatively small sample sizes, our approach demonstrates the inference potential to be gained from detailed kinship studies, and highlights the importance of collecting long-term genetic data in gaining a better understanding of the ecology of elusive long-lived animals.

## Electronic supplementary material

ESM 1 (DOCX 60 kb)
