# Supplementary material for: Temporal and geographic patterns of kinship structure in common dolphins (Delphinus delphis) suggest site fidelity and female-biased long-distance dispersal
Source: Behav Ecol Sociobiol. 2017 Jul 21;71(8):123. doi: 10.1007/s00265-017-2351-z (PMC5522516; doi:10.1007/s00265-017-2351-z)
Supplement: Supplementary file 1 — (DOCX 60 kb) [file 265_2017_2351_MOESM1_ESM.docx]

**Behavioral Ecology and Sociobiology**

**Supplementary Material for the manuscript:** Temporal and geographic patterns of kinship structure in common dolphins (*Delphinus delphis*) suggest site fidelity and female-biased long distance dispersal

*Laura Ball^1*^, Kypher Shreves^1*^, Małgorzata Pilot^1^, André E. Moura^1^*

*^1^ School of Life Sciences, University of Lincoln, Lincoln, UK*

** These authors contributed equally to the study*

*Corresponding author: Andre E. Moura,* [*amoura@lincoln.ac.uk*](mailto:amoura@lincoln.ac.uk)*, Tel: +44 (0)1522886805*


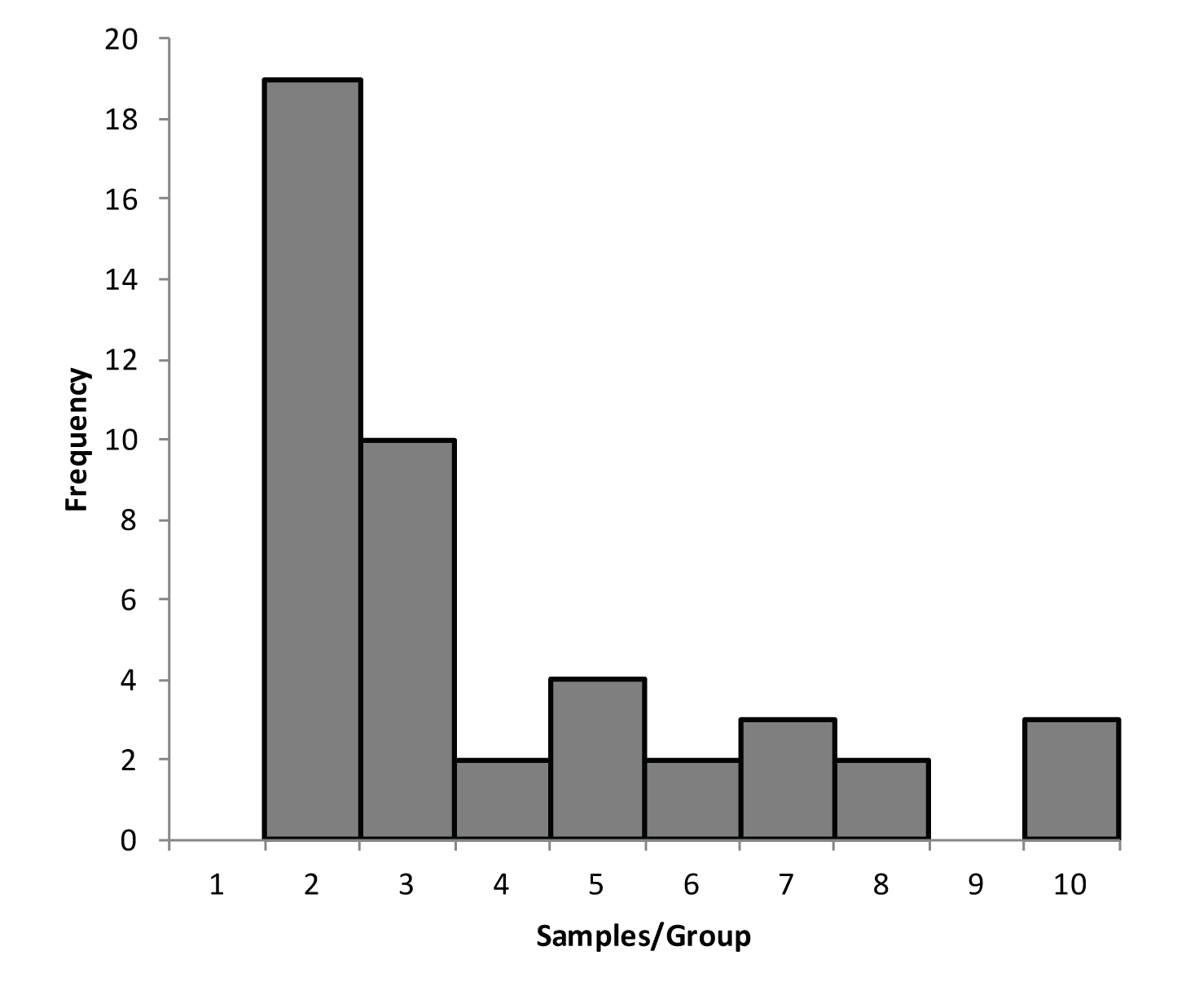


Figure S1. Frequency distribution of sample number per group used in this study.

Table S1. Full-sibling pairs matched with CERVUS parent-offspring pairs

| **Full Siblings Group** | **Identified as Parent and Offspring by CERVUS** | **Parent- Offspring** |
| --- | --- | --- |
| 1 | N | - |
| 2 | N | - |
| 3 | N | - |
| 4 | Y | Father-Offspring |
| 5 | N | - |
| 6 | N | - |
| 7 | N | - |
| 8 | N | - |
| 9 | Y | Mother- Offspring |
|  |  |  |
| 10 | N | - |
| 11 | N | - |
| 12 | N | - |
| 13 | Y | Mother- Offspring |
|  |  |  |
| 14 | N | - |
| 15 | Y | Father-Offspring |
| 16 | Y | Mother- Offspring |
| 17 | Y | Mother- Offspring |
|  |  |  |
| 18 | N | - |
| 19 | N | - |
| 20 | N | - |
| 21 | N | - |
| 22 | N | - |

Table S2. Cousin raw data; location, sample date, sample time.

| **Cousins Group Number** | **ID** | **Date** | **Time** | **Seconds** | **Region Sampled** |
| --- | --- | --- | --- | --- | --- |
| 1 | Dd2-D10 | 12-Jul-09 | 1101 | 58 | Figueira |
|  | Dd-J1 | 16-Aug-08 | 1335 | 36 | Peniche |
| 2 | Dd-I2 | 15-Aug-08 | 1538 | 54 | Peniche |
|  | Dd2-F2 | 27-Jul-09 | 1000 | 46 | Sines |
| 3 | Dd2-C8 | 24-Jun-09 | 1736 | 49 | Porto |
|  | Dd2-E7 | 16-Jul-09 | 1520 | 23 | Figueira |
|  | Dd2-F5 | 30-Jul-09 | 1151 | 59 | Sines |
|  | Dd2-F6 | 30-Jul-09 | 1208 | 27 | Sines |
|  | Dd-B10 | 05-Aug-07 | 0945 | 48 | Sagres |
| 4 | Dd2-E10 | 16-Jul-09 | 1601 | 25 | Figueira |
|  | Dd-I10 | 17-Aug-08 | 1436 | 24 | Peniche |
|  | Dd2-A3 | 17-Aug-08 | 1537 | 56 | Peniche |
|  | Dd2-C1 | 28-Aug-08 | 1758 | 42 | Sines |
|  | Dd-C1 | 05-Aug-07 | 0957 | 8 | Sagres |
|  | Dd-C6 | 05-Aug-07 | 1051 | 3 | Sagres |
| 5 | Dd-F4 | 15-Jul-08 | 1046 | 38 | Porto |
|  | Dd2-C10 | 24-Jun-09 | 1751 | 14 | Porto |
|  | Dd-H8 | 15-Aug-08 | 1433 | 46 | Peniche |
|  | Dd-I7 | 16-Aug-08 | 1218 | 50 | Peniche |
|  | Dd2-F4 | 30-Jul-09 | 1133 | 30 | Sines |
|  | Dd2-F7 | 30-Jul-09 | 1231 | 12 | Sines |
|  | Dd-B5 | 03-Aug-07 | 1004 | 19 | Sagres |
|  | Dd-C2 | 05-Aug-07 | 1013 | 8 | Sagres |
|  | Dd-C8 | 11-Aug-07 | 1208 | 53 | Sagres |
|  | Dd2-H10 | 11-Aug-09 | 1556 | 55 | Sagres |
| 6 | Dd-F9 | 27-Jul-08 | 1216 | 35 | Figueira |
|  | Dd-G9 | 11-Aug-08 | 1110 | 39 | Figueira |
|  | Dd2-E2 | 16-Jul-09 | 1255 | 9 | Figueira |
|  | Dd-J2 | 16-Aug-08 | 1341 | 39 | Peniche |
|  | Dd2-I6 | 13-Aug-09 | 1246 | 46 | Sagres |
|  | Dd2-J1 | 13-Aug-09 | 1345 | 58 | Sagres |
| 7 | Dd-E10 | 07-Jul-08 | 1215 | 56 | Porto |
|  | Dd2-C5 | 24-Jun-09 | 1519 | 19 | Porto |
|  | Dd2-C7 | 24-Jun-09 | 1628 | 4 | Porto |
|  | Dd2-D1 | 24-Jun-09 | 1807 | 3 | Porto |
|  | Dd2-D2 | 24-Jun-09 | 1813 | 49 | Porto |
|  | Dd-D3 | 30-Jul-08 | 1523 | 9 | Figueira |
|  | Dd-I8 | 16-Aug-08 | 1224 | 47 | Peniche |
|  | Dd-I3 | 16-Aug-08 | 1354 | 34 | Peniche |
|  | Dd2-G3 | 01-Aug-09 | 1007 | 39 | Sagres |
|  | Dd2-G5 | 01-Aug-09 | 1402 | 1 | Sagres |
|  | Dd2-G7 | 04-Aug-09 | 1038 | 43 | Sagres |
|  | Dd-A3 | 24-Jul-07 | 1216 | 26 | Portimao |
| 8 | Dd-I1 | 15-Aug-08 | 1516 | 39 | Peniche |
|  | Dd-J7 | 17-Aug-08 | 1331 | 4 | Peniche |
|  | Dd-A1 | 24-Jul-07 | 1154 | 11 | Portimao |
|  | Dd-D8 | 10-Jun-08 | 1601 | 55 | Portimao |
| 9 | Dd-F6 | 15-Jul-08 | 1157 | 13 | Porto |
|  | Dd2-14 | 11-Aug-09 | 1646 | 12 | Sagres |
|  | Dd2-G10 | 06-Aug-09 | 1228 | 9 | Portimao |
| 10 | Dd-J4 | 17-Aug-08 | 1208 | 51 | Peniche |
|  | Dd2-B3 | 27-Aug-08 | 1451 | 40 | Sines |
|  | Dd2-C3 | 28-Aug-08 | 1816 | 57 | Sines |
|  | Dd-B1 | 27-Jul-07 | 1402 | 8 | Portimao |
|  | Dd2-H3 | 06-Aug-09 | 1251 | 0 | Portimao |
| 11 | Dd-G1 | 30-Jul-08 | 1119 | 53 | Figueira |
|  | Dd-D8 | 10-Aug-08 | 1356 | 18 | Figueira |
|  | Dd-G10 | 11-Aug-08 | 1126 | 7 | Figueira |
|  | Dd2-E1 | 12-Jul-09 | 1126 | 19 | Figueira |
|  | Dd2-H9 | 11-Aug-09 | 1548 | 3 | Sagres |
|  | Dd2-I3 | 11-Aug-09 | 1632 | 33 | Sagres |
|  | Dd-D7 | 10-Jun-08 | 1338 | 6 | Portimao |
|  | Dd-E3 | 12-Jun-08 | 1750 | 39 | Portimao |
|  | Dd2-J4 | 16-Aug-09 | 1417 | 45 | Portimao |
| 12 | Dd2-D4 | 25-Jun-09 | 1754 | 16 | Porto |
|  | Dd-F7 | 24-Jul-08 | 1410 | 39 | Figueira |
|  | Dd-02 | 30-Jul-08 | 1304 | 25 | Figueira |
|  | Dd-D5 | 31-Jul-08 | 1025 | 2 | Figueira |
|  | Dd-D6 | 07-Aug-08 | 1451 | 51 | Figueira |
|  | Dd-H2 | 12-Aug-08 | 1009 | 47 | Figueira |
|  | Dd-C10 | 09-Sep-07 | 1130 | 36 | Sines |
|  | Dd2-B4 | 28-Aug-08 | 1553 | 39 | Sines |
|  | Dd-C3 | 05-Aug-07 | 1022 | 50 | Sagres |
|  | Dd-A6 | 25-Jul-07 | 1121 | 1 | Portimao |
|  | Dd2-H6 | 09-Aug-09 | 1206 | 41 | Portimao |
|  | Dd2-J5 | 16-Aug-09 | 1424 | 23 | Portimao |
| 13 | Dd-F1 | 09-Jul-08 | 1446 | 36 | Porto |
|  | Dd2-D5 | 03-Jul-09 | 1703 | 36 | Porto |
|  | Dd2-D9 | 04-Jul-09 | 1802 | 22 | Porto |
|  | Dd-F8 | 27-Jul-08 | 1205 | 26 | Figueira |
|  | Dd-G7 | 07-Aug-08 | 1501 | 9 | Figueira |
|  | Dd-J5 | 17-Aug-08 | 1222 | 4 | Peniche |
|  | Dd2-A4 | 24-Aug-08 | 1303 | 57 | Sines |
|  | Dd2-B8 | 28-Aug-08 | 1732 | 6 | Sines |
|  | Dd2-B10 | 28-Aug-08 | 1748 | 28 | Sines |
|  | Dd2-G1 | 30-Jul-09 | 1342 | 7 | Sines |
|  | Dd-C5 | 05-Aug-07 | 1040 | 46 | Sagres |
|  | Dd-D2 | 16-Sep-07 | 0952 | 30 | Sagres |
|  | Dd2-J3 | 14-Aug-09 | 1453 | 29 | Sagres |
|  | Dd-A5 | 24-Jul-07 | 1241 | 15 | Portimao |
|  | Dd2-G9 | 05-Aug-09 | 1630 | 14 | Portimao |
|  | Dd2-H2 | 06-Aug-09 | 1242 | 46 | Portimao |
|  | Dd2-H5 | 06-Aug-09 | 1409 | 49 | Portimao |
|  | Dd2-J6 | 16-Aug-09 | 1437 | 41 | Portimao |
|  | Dd2-J7 | 16-Aug-09 | 1542 | 7 | Portimao |
| 14 | Dd-I4 | 16-Aug-08 | 1058 | 8 | Peniche |
|  | Dd-J6 | 17-Aug-08 | 1236 | 45 | Peniche |
|  | Dd2-B6 | 28-Aug-08 | 1626 | 12 | Sines |
|  | Dd2-I2 | 11 -Aug-09 | 1625 | 56 | Sagres |
|  | Dd2-I10 | 13-Aug-09 | 1333 | 46 | Sagres |
|  | Dd2-J2 | 13-Aug-09 | 1400 | 12 | Sagres |
|  | Dd-A4 | 24-Jul-07 | 1234 | 28 | Portimao |
|  | Dd-E4 | 14-Jun-08 | 1420 | 19 | Portimao |
|  | Dd2-J8 | 16-Aug-09 | 1549 | 36 | Portimao |
| 15 | Dd-E6 | 03-Jul-08 | 1319 | 50 | Porto |
|  | Dd-F5 | 15-Jul-08 | 1148 | 20 | Porto |
|  | Dd2-D7 | 03-Jul-09 | 1853 | 34 | Porto |
|  | Dd2-F1 | 16-Jul-09 | 1623 | 4 | Figueira |
|  | Dd-J8 | 17-Aug-08 | 1350 | 4 | Peniche |
|  | Dd2-B7 | 28-Aug-08 | 1643 | 23 | Sines |
|  | Dd-A7 | 25-Jul-07 | 1148 | 2 | Portimao |
|  | Dd2-J9 | 16-Aug-09 | 1601 | 5 | Portimao |
| 16 | Dd-H1 | 11-Aug-08 | 1155 | 9 | Figueira |
|  | Dd-C9 | 09-Sep-07 | 1116 | 30 | Sines |
|  | Dd2-J10 | 16-Aug-09 | 1612 | 9 | Portimao |
| 17 | Dd-H3 | 12-Aug-08 | 1015 | 40 | Figueira |
|  | Dd2-E3 | 16-Jul-09 | 1428 | 15 | Figueira |
|  | Dd-H9 | 15-Aug-08 | 1441 | 59 | Peniche |
|  | Dd-H10 | 15-Aug-08 | 1457 | 17 | Peniche |
|  | Dd-I9 | 16-Aug-08 | 1252 | 29 | Peniche |
|  | Dd2-I8 | 13-Aug-09 | 1307 | 29 | Sagres |
|  | Dd-A9 | 26/27-Jul-07 | 0 | 0 | Portimao |
|  | Dd-D5 | 10-Jun-08 | 1258 | 41 | Portimao |
|  | Dd3-A1 | 18-Aug-09 | 1306 | 43 | Portimao |
|  | Dd3-A3 | 18-Aug-09 | 1341 | 59 | Portimao |
| 18 | Dd-F3 | 14-Jul-08 | 1251 | 27 | Porto |
|  | Dd2-E6 | 16-Jul-09 | 1515 | 3 | Figueira |
|  | Dd-I9 | 17-Aug-08 | 1415 | 14 | Peniche |
|  | Dd2-A9 | 27-Aug-08 | 1349 | 43 | Sines |
|  | Dd-B7 | 03-Aug-07 | 1048 | 24 | Sagres |
|  | Dd-B9 | 05-Aug-07 | 0933 | 32 | Sagres |
|  | Dd-C4 | 05-Aug-07 | 1032 | 40 | Sagres |
|  | Dd-C7 | 09-Aug-07 | 1229 | 53 | Sagres |
|  | Dd2-H8 | 11 -Aug-09 | 1540 | 42 | Sagres |
|  | Dd2-I9 | 13-Aug-09 | 1321 | 14 | Sagres |
|  | Dd-D10 | 10-Jun-08 | 1625 | 34 | Portimao |
|  | Dd2-H4 | 06-Aug-09 | 1351 | 57 | Portimao |
|  | Dd3-A4 | 18-Aug-09 | 1449 | 8 | Portimao |
| 19 | Dd-G4 | 30-Jul-08 | 1625 | 22 | Figueira |
|  | Dd-H6 | 15-Aug-08 | 1158 | 50 | Peniche |
|  | Dd-I5 | 16-Aug-08 | 1131 | 30 | Peniche |
|  | Dd2-A10 | 27-Aug-08 | 1411 | 7 | Sines |
|  | Dd2-F8 | 30-Jul-09 | 1238 | 13 | Sines |
|  | Dd2-G4 | 01-Aug-09 | 1047 | 39 | Sagres |
|  | Dd2-I1 | 11-Aug-09 | 1614 | 7 | Sagres |
|  | Dd-A8 | 26-Jul-07 | 1333 | 22 | Portimao |
|  | Dd3-A2 | 18-Aug-09 | 1324 | 46 | Portimao |
|  | Dd3-A7 | 19-Aug-09 | 1417 | 34 | Portimao |
| 20 | Dd2-E8 | 16-Jul-09 | 1532 | 28 | Figueira |
|  | Dd2-A8 | 27-Aug-08 | 1248 | 41 | Sines |
|  | Dd2-B5 | 28-Aug-08 | 1614 | 42 | Sines |
|  | Dd2-F9 | 30-Jul-09 | 1249 | 12 | Sines |
|  | Dd2-F10 | 30-Jul-09 | 1327 | 20 | Sines |
|  | Dd-B6 | 03-Aug-07 | 1009 | 21 | Sagres |
|  | Dd3-A8 | 19-Aug-09 | 1446 | 8 | Portimao |
| 21 | Dd2-D3 | 24-Jun-09 | 1838 | 50 | Porto |
|  | Dd-H7 | 15-Aug-08 | 1234 | 7 | Peniche |
|  | Dd-I3 | 16-Aug-08 | 1027 | 30 | Peniche |
|  | Dd-I6 | 16-Aug-08 | 1154 | 20 | Peniche |
|  | Dd-B8 | 03-Aug-07 | 1031 | 36 | Sagres |
|  | Dd-D3 | 16-Sep-07 | 1003 | 7 | Sagres |
|  | Dd-A10 | 27-Jul-07 | 1359 | 27 | Portimao |
|  | Dd3-A6 | 19-Aug-09 | 1405 | 35 | Portimao |
|  | Dd3-A9 | 19-Aug-09 | 1506 | 41 | Portimao |
| 22 | Dd-E5 | 03-Jul-08 | 1255 | 56 | Porto |
|  | Dd-E7 | 06-Jul-08 | 1046 | 4 | Porto |
|  | Dd-F2 | 09-Jul-08 | 1450 | 33 | Porto |
|  | Dd2-A6 | 24-Aug-08 | 1348 | 43 | Sines |
|  | Dd2-A7 | 27-Aug-08 | 1226 | 41 | Sines |
|  | Dd2-B2 | 27-Aug-08 | 1439 | 36 | Sines |
|  | Dd2-C2 | 28-Aug-08 | 1808 | 37 | Sines |
|  | Dd-D1 | 16-Sep-07 | 0921 | 44 | Sagres |
|  | Dd3-A5 | 18-Aug-09 | 1501 | 35 | Portimao |
|  | Dd3-A10 | 19-Aug-09 | 1535 | 17 | Portimao |
| 23 | Dd-E9 | 07-Jul-08 | 1207 | 14 | Porto |
|  | Dd2-C9 | 24-Jun-09 | 1741 | 53 | Porto |
|  | Dd2-D8 | 03-Jul-09 | 1901 | 24 | Porto |
|  | Dd-I10 | 16-Aug-08 | 1322 | 43 | Peniche |
|  | Dd2-A2 | 17-Aug-08 | 1531 | 14 | Peniche |
|  | Dd2-B1 | 27-Aug-08 | 1425 | 0 | Sines |
|  | Dd2-B9 | 28-Aug-08 | 1735 | 39 | Sines |
|  | Dd-083 | 03-Aug-07 | 0942 | 21 | Sagres |
|  | Dd2-G6 | 04-Aug-09 | 1028 | 13 | Sagres |
|  | Dd-D4 | 26-Sep-07 | 1426 | 37 | Portimao |
|  | Dd-D9 | 10-Jun-08 | 1636 | 50 | Portimao |
|  | Dd-E1 | 11-Jun-08 | 1618 | 13 | Portimao |
|  | Dd-E2 | 12-Jun-08 | 1646 | 43 | Portimao |
|  | Dd3-B3 | 20-Aug-09 | 1117 | 48 | Portimao |
| 24 | Dd-E8 | 07-Jul-08 | 1200 | 56 | Porto |
|  | Dd2-E9 | 16-Jul-09 | 1545 | 57 | Figueira |
|  | Dd2-A1 | 17-Aug-08 | 1511 | 33 | Peniche |
|  | Dd-A2 | 24-Jul-07 | 1209 | 39 | Portimao |
|  | Dd2-H7 | 09-Aug-09 | 1219 | 24 | Portimao |
|  | Dd3-B4 | 20-Aug-09 | 1124 | 22 | Portimao |
| 25 | Dd2-C4 | 19-Jun-09 | 1512 | 52 | Porto |
|  | Dd-H5 | 15-Aug-08 | 1152 | 6 | Peniche |
|  | Dd2-F3 | 27-Jul-09 | 1254 | 42 | Sines |
|  | Dd-B4 | 03-Aug-07 | 0955 | 41 | Sagres |
|  | Dd2-G2 | 01-Aug-09 | 0949 | 15 | Sagres |
|  | Dd2-I5 | 13-Aug-09 | 1241 | 45 | Sagres |
|  | Dd-B2 | 27-Jul-07 | 1444 | 52 | Portimao |
|  | Dd-D6 | 10-Jun-08 | 1305 | 31 | Portimao |
|  | Dd2-G8 | 05-Aug-09 | 1615 | 4 | Portimao |
|  | Dd3-B5 | 20-Aug-09 | 1130 | 31 | Portimao |
|  | | | | | |

Table S3 - Results from sex-biased dispersal tests, carried out in Fstat. mAIc - mean Assignment Index; vAIc - variance of Assignment Index.

|  | *F*_IS_ | | | *F*_ST_ | | | mAIc | | vAIc | |
| --- | --- | --- | --- | --- | --- | --- | --- | --- | --- | --- |
|  | M | F | Overall | M | F | Overall | M | F | M | F |
| Test Value | 0.0235 | 0.063 | 0.0168 | 0.0015 | 0.0002 | 0.0014 | -0.0219 | 0.0319 | 12.42 | 10.86 |
| p-Value | 0.7660 | | | 0.283 | | | 0.5620 | | 0.7590 | |
